# Structure of an Amino Acid-Decorated Exopolysaccharide Secreted by a *Vibrio alginolyticus* Strain

**DOI:** 10.3390/md13116723

**Published:** 2015-10-30

**Authors:** Sophie Drouillard, Isabelle Jeacomine, Laurine Buon, Claire Boisset, Anthony Courtois, Bertrand Thollas, Pierre-Yves Morvan, Romuald Vallée, William Helbert

**Affiliations:** 1 Université Grenoble Alpes, CERMAV, CNRS, 38000 Grenoble, France; E-Mails: sophie.drouillard@cermav.cnrs.fr (S.D.); isabelle.jeacomine@cermav.cnrs.fr (I.J.); laurine.buon.drouillard@cermav.cnrs.fr (L.B.); claire.boisset-helbert@cermav.cnrs.fr (C.B.); 2 Polymaris Biotechnology, Aéropôle Centre, 29600 Morlaix, France; E-Mails: Anthony.courtois@polymaris.com (A.C.); Bertrand.thollas@polymaris.com (B.T.); 3 Codif International, 35610 Roz-sur-Couesnon, France; E-Mails: py.morvan@codif.com (P.-Y.M.); r.vallee@codif.com (R.V.)

**Keywords:** exopolysaccharide, *Vibrio alginolyticus*, amino acids, ingredient

## Abstract

*Vibrio alginolyticus* (CNCM I-4994) secretes an exopolysaccharide that can be used as an ingredient in cosmetic applications. The structure was resolved using chromatography and one- and two-dimensional NMR spectroscopy experiments. The results show that the carbohydrate backbone is made of two residues: d-galacturonic acid and *N*-acetyl-d-glucosamine (GlcNac), which together constitute a tetrasaccharide repetition unit: [→3)-α-d-GalA-(1→4)-α-d-GalA-(1→3)-α-d-GalA-(1→3)-β-GlcNAc(1→]. Two amino acids, alanine and serine, are linked to GalA residues via amido linkages. The position and the distribution of the amino acids were characterized by two-dimensional NMR spectroscopy. To our knowledge, this is the first description of a structure for a marine exopolysaccharide decorated with an amino acid.

## 1. Introduction

Many marine bacteria secrete extracellular polysaccharides, called exopolysaccharides (EPSs), which constitute an immense reservoir of novel molecules whose structure and functional properties are largely unknown. The structural diversity of EPSs—as for all other polysaccharides—lies in the stereochemistry of the carbohydrate residues, the glycosidic linkage between the residues, and, in turn, the identity of the (oligo)saccharide repetition units. Homopolysaccharides have the simplest structure, being composed of one type of carbohydrate residue; heteropolysaccharides are generally composed of 2 to 8 different residues, with some exceptionally highly complex structures containing up to 10 to 12 different residues. A second level of EPS complexity is provided by the decoration of the carbohydrate backbone with organic (e.g., acetate, pyruvate) or inorganic (e.g., sulfate ester) derivatives [1].

The structural diversity of marine EPSs is associated with a wide range of biological and physico-chemical properties. EPSs create a micro-environment that facilitates bacterial reproduction and protects bacterial cells from strong environmental variation. Other functions attributed to EPSs include attachment of bacterial cells to surfaces, the formation of biofilms, as well as the concentration or sequestration of specific molecules (e.g., signaling, nutriments) [2]. *In vitro*, several marine EPSs have been shown to have biological activity, including anti-tumor activity, immunostimulatory activity, anticomplementary activity, and involvement in bone and tissue regeneration [3,4,5]. Although marine EPSs have aroused considerable interest, their use in industry is still very limited in contrast to, for example, terrestrial bacterial EPSs, such as xanthan (*Xanthomonas campestris*) or gellan (*Sphingomonas* *paucimobilis*) which are employed in the food industry for their viscosifying and gelling properties.

Production costs and compliance with regulations hamper the industrial-scale use of marine EPSs as hydrocolloids for food applications, and competition with the already marketed plant and algal polysaccharides is currently insurmountable [1,6]. However, the production of marine EPSs has several technical advantages: their production can be easily controlled and is independent of seasonal variation and they can be very highly purified, making them appropriate for niche applications in the biomedical and cosmetics sectors. One example is the EPS secreted by the Gram-negative marine bacteria *Vibrio alginolyticus* (CNCM I-4994), which is produced on an industrial scale as an ingredient for cosmetic applications for its matifying and anti-inflammatory properties [7]. This strain was isolated from deep water in the Aber Wrac’h estuary (Brittany, France). It lacks the *tdh* and *trh* genes coding for thermostable direct hemolysin (TDH) and/or TDH-related hemolysin (TRH), respectively, involved in pathogenicity of numerous *Vibrio* strains [8]. Here, we report the chemical structure of *V. alginolyticus* EPS (VA-EPS), which has a very original glucosaminogalactan backbone decorated with alanine and serine amino acids.

## 2. Results and Discussion

### 2.1. Purification and Composition of the EPSs

The EPS was purified in several steps of filtration and precipitation with ethanol. The Bradford assay indicated the absence of any detectable proteins, thereby attesting to the high purity of the polysaccharides. However, alanine and serine were detected in high-performance liquid chromatographs after complete hydrolysis of the sample, suggesting that they were not free amino acids. Size-exclusion chromatography (SEC) coupled to multi-angle laser light scattering confirmed that the purified EPS was likely composed of one species of molecule having a molecular weight of 1.16 × 10^6^ Da with a narrow polydispersity index of 1.34.

Composition analysis using gas-chromatography based on the trimethysilyl method on the residues obtained after complete hydrolysis of the polysaccharides revealed only two residues: galacturonic acid (GalA) and *N*-acetyl-glucosamine (GlcNAc) in a ratio of about 3.0:1.0 GalA:GlcNAc. Elementary analysis did not uncover any inorganic derivatives such as sulfate or phosphate ester groups. Methylation analyses, shown in Table 1, indicated that the GalA residues were involved either in 1,3-, 1,4- or 1,3,6-linkages and the GlcNAc residue in 1,3-linkages.

**Table 1:** Methylation analysis of the *Vibrio alginolyticus* exopolysaccharide (VA-EPS) after carboxyl reduction.

Type of Sugar	Partially Methylated Alditol Acetate	Deduced Linkage
Galactose	1,3,5-Tri-*O*-acetyl-1-deuterio-2,4,6-tri-*O*-methyl-d-Galactitol	→3)-Gal*p*-(1→
	1,4,5-Tri-*O*-acetyl-1-deuterio-2,3,6-tri-*O*-methyl-d-Galactitol	→4)-Gal*p*-(1→
	1,3,5,6-Tetra-*O*-acetyl-1-deuterio-3,4-di-*O*-methyl-d-Galactitol	→3,6)-Gal*p*-(1→
*N*-acetyl Glucosamine	1,3,5-Tri-*O*-acetyl-2-(acetylmethylamino)-2-deoxy-1-deuterio-4,6-di-*O*-methyl-d-Galactitol	→3)-GlcNAc-(1→

**Figure 1:**
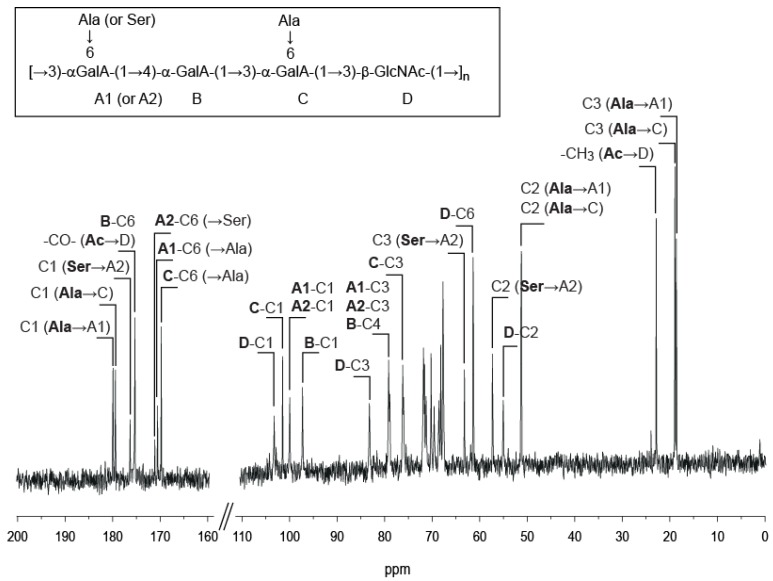
^13^C NMR of *Vibrio alginolyticus* exopolysaccharide recorded at 353 K. Inset: chemical structure of the polysaccharide.

The ^13^C NMR spectrum of the polysaccharide (Figure 1) showed signals at 95–105 ppm and were attributed to four anomeric carbons. Three signals were observed in the region of the methyl groups (15–25 ppm): the first corresponded to the methyl group of an acetyl group (22.68 ppm) and the two other signals (18.44 and 18.91 ppm) were attributed to methyl groups of amino acids. Eight different signals were observed in the region of the carbonyl groups between 175 and 180 ppm and likely corresponded to C6 of free uronic acids, carboxyl groups of amino acids and amide groups.

**Figure 2:**
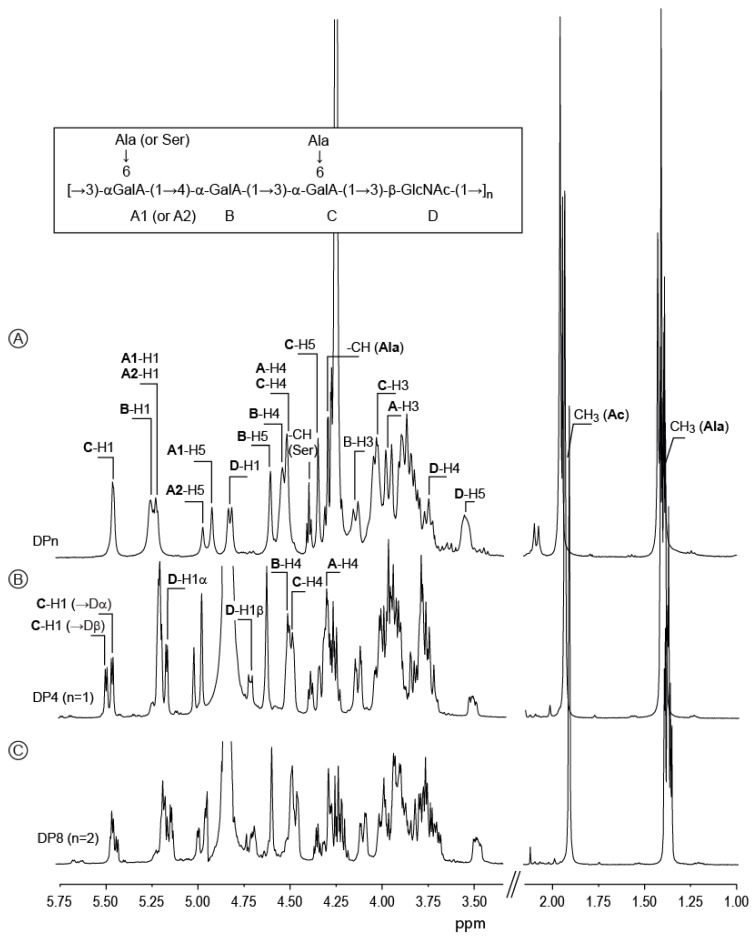
^1^H NMR of the *Vibrio alginolyticus* exopolysaccharide and its oligosaccharides (DP4, DP8) purified after acid hydrolysis. (**A**) The ^1^H NMR of the polysaccharide was recorded at 353 K; (**B**,**C**) ^1^H NMR of DP4 and DP8, respectively, recorded at 293 K. Inset: chemical structure of the polysaccharide.

Inspection of ^1^H NMR spectra (Figure 2) and ^1^H/^13^C heteronuclear single-quantum correlations (HSQC) of the EPS (not shown) confirmed a repetition unit made of four distinct residues of which three (A–C) adopt an α-anomeric configuration and the fourth (D) has a β-anomeric configuration. ^1^H NMR spectra confirmed that the repetition moiety is a tetrasaccharide decorated with *N*-acetyl groups and amino acids.

### 2.2. Detailed Structure Analysis of the EPS Using NMR

We assigned the ring protons of the three α-anomeric residues (A–C) starting from the anomeric protons A-H1, B-H1 and C-H1 by successfully combining correlation spectroscopy (COSY) and total COSY (TOCSY) analyses (Table 2). The chemical shifts of the corresponding carbons were determined using ^1^H/^13^C heteronuclear single-quantum correlation (HSQC) and are reported in Table 3.

**Table 2:** Proton chemical shifts (ppm) of the *Vibrio alginolyticus* exopolysaccharide (VA-EPS) and the purified oligosaccharides (DP4, DP8). A–D refer to the residues composing the repetition moieties of the polysaccharide. When the residues are located at the reducing end or at the non-reducing end, they are labelled r and nr, respectively.

	VA-EPS: [ABCD]_n_	DP4: A^nr^BC^r′^D^r^	DP8: A^nr^BCDABC^r′^D^r^
A1: 6Ala-GalA/A2:6Ser-GalA			
H1	5.18/5.19		5.20/5.20
H2	3.84/3.84		3.80/3.80
H3	4.00/4.00		4.04/4.04
H4	4.49/4.51		4.54/4.54
H5	4.91/4.96		4.99/5.04
A1^nr^: 6Ala-GalA/A2^nr^:6Ser-GalA			
H1		5.22/5.23	5.20/5.22
H2		3.78/3.78	3.80/3.80
H3		3.97/3.99	3.99/3.99
H4		4.33/4.36	4.34/4.34
H5		4.99/5.04	4.99/5.04
B: GalA			
H1	5.22	5.22	5.23
H2	3.93	3.93	3.95
H3	4.10	4.14	4.15
H4	4.51	4.52	4.53
H5	4.58	4.64	4.65
C: 6Ala-GalA			
H1	5.43		5.50
H2	3.97		3.98
H3	4.03		4.02
H4	4.48		4.50
H5	4.31		4.32
C^r′^: 6Ala-GalA			
H1-D^r^ α/β		5.48/5.51	5.48/5.51
H2-D^r^ α/β		3.98/3.97	3.98/3.97
H3-D^r^ α/β		4.02/4.00	4.03/4.00
H4		4.50	4.50
H5		4.31	4.32
D: GlcNAc			
H1	4.77		4.75
H2	3.85		3.87
H3	3.81		3.87
H4	3.70		3.75
H5	3.51		3.53
H6′	3.93		3.95
H6′	3.77		3.82
NHAc:CH_3_	1.95		1.95
D^r^: GlcNAc			
H1 α/β		5.18/4.73	5.18/4.73
H2 α/β		4.03/3.80	4.03/3.81
H3 α/β		3.99/3.81	4.00/3.81
H4 α/β		3.75/3.73	3.75/3.73
H5 α/β		3.92/3.52	3.93/3.53
H6 α/β		3.84/3.91	3.95/3.82
H6′		3.78	3.82
NHAc: CH_3_		1.95	1.95

**Table 3:** Carbon chemical shifts (ppm) of the *Vibrio alginolyticus* exopolysaccharide (VA-EPS) and the purified oligosaccharides (DP4, DP8).

	VA-EPS: [ABCD]_n_	DP4: A^nr^BC^r′^D^r^	DP8: A^nr^BCDABC^r′^D^r^
A1: 6Ala-GalA/A2: 6Ser-GalA			
C1	99.82/99.94		100.07/100.22
C2	67.99/67.99		68.81/68.81
C3	78.97/78.97		79.14/79.23
C4	70.12/70.12		70.34/70.34
C5	71.43/71.60		71.92/72.02
C6	170.50/171.05		170.94/171.37
A1^nr^: 6Ala-GalA/A2^nr^: 6Ser-GalA			
C1		100.07/100.22	100.07/100.22
C2		68.84/68.84	68.81/68.81
C3		69.78/69.78	69.76/69.76
C4		70.47/70.43	70.47/70.43
C5		71.93/72.04	71.92/72.04
C6		171.10/171.54	171.10/171.54
B: GalA			
C1	97.20	97.63	97.66
C2	68.33	68.34	68.34
C3	69.34	69.25	69.25
C4-A1/A2	78.68/78.85	78.93/79.15	78.80/78.93
C5	71.82	71.73	71.73
C6	175.34	175.80	175.83
C: 6Ala-GalA			
C1	101.32		101.06
C2	67.40		67.27
C3	76.06		76.37
C4	67.61		67.92
C5	71.72		71.66
C6	169.68		170.05
C^r′^: 6Ala-GalA			
C1-D^r^ α/β		101.39/101.04	101.37/101.06
C2-D^r^ α/β		67.28/67.44	67.27/67.44
C3-D^r^ α/β		76.41/76.36	76.37/76.26
C4-D^r^ α/β		67.90/67.84	67.92/67.86
C5-D^r^ α/β		71.67/71.73	71.51/71.66
C6-D^r^ α/β		170.01/170.14	170.05/170.15
D: GlcNAc			
C1	103.17		103.55
C2	54.84		54.85
C3	82.89		81.74
C4	71.03		70.93
C5	75.87		75.93
C6	61.13		60.91
NHAc: CH_3_/CO	22.68/175.25		22.76/175.64
D^r^: GlcNAc			
C1 α/β		91.76/95.73	91.76/95.73
C2 α/β		53.11/55.80	53.11/55.80
C3 α/β		80.37/81.89	80.37/81.90
C4 α/β		71.09	71.08
C5 α/β		72.15/76.28	72.15/76.26
C6 α/β		61.02/61.17	61.01/61.14
NHAc: CO		174.90/175.20	174.88/175.19
NHAc: CH_3_		22.44/22.70	22.46/22.72

The chemical shifts as well as the coupling constants were in agreement with three α-d-galacturonic residues. For example, the A-H4, B-H4 and C-H4 protons observed at about 4.50 ppm were characteristic of the chemical shifts observed for uronic residues. Detailed inspection of the coupling of the H5 protons with C6 carboxyl groups or amide groups by ^1^H/^13^C heteronuclear multiple-bond correlation (HMBC) revealed that there were four instead of three α-d-galacturonic residues. The A-H5 protons (A1-H5: 4.91, A2-H5: 4.96 ppm) correlated with two carbon signals corresponding to two different amide groups (A1-C6: 170.50 ppm, A2-C6: 171.05 ppm) suggesting that these residues were likely bound to two different amino acids (see detailed analyses below). The B-H5 proton (4.58 ppm) was coupled to the carbon of a free carbonyl group (B-C6: 175.34 ppm), indicating that the B residue is an underived α-d-GalA. The C-H5 proton (4.31 ppm) coupled to a single carbon (C-C6: 169.68 ppm) belonged to an amide group.

Assignment of the protons and carbons of the D residue was conducted similarly using COSY, TOCSY and HSQC analyses. The chemical shifts reported in Table 2 and Table 3 were in agreement with a β-GlcNH (or β-GlcNAc) residue with in particular the characteristic D-H5 proton observed at 3.51 ppm. Coupling of the nitrogen (D-NAc) on the acetyl group with D-H1 (4.77 ppm) and D-H2 (3.85 ppm) revealed by rotating frame nuclear Overhauser effect spectroscopy (ROESY) experiments confirmed that the acetyl group is bound to the β-d-GlcNH. Proton NMR and ROESY experiments suggested that all the β-d-GlcNH were acetylated, giving only β-d-GlcNAc residues.

**Figure 3:**
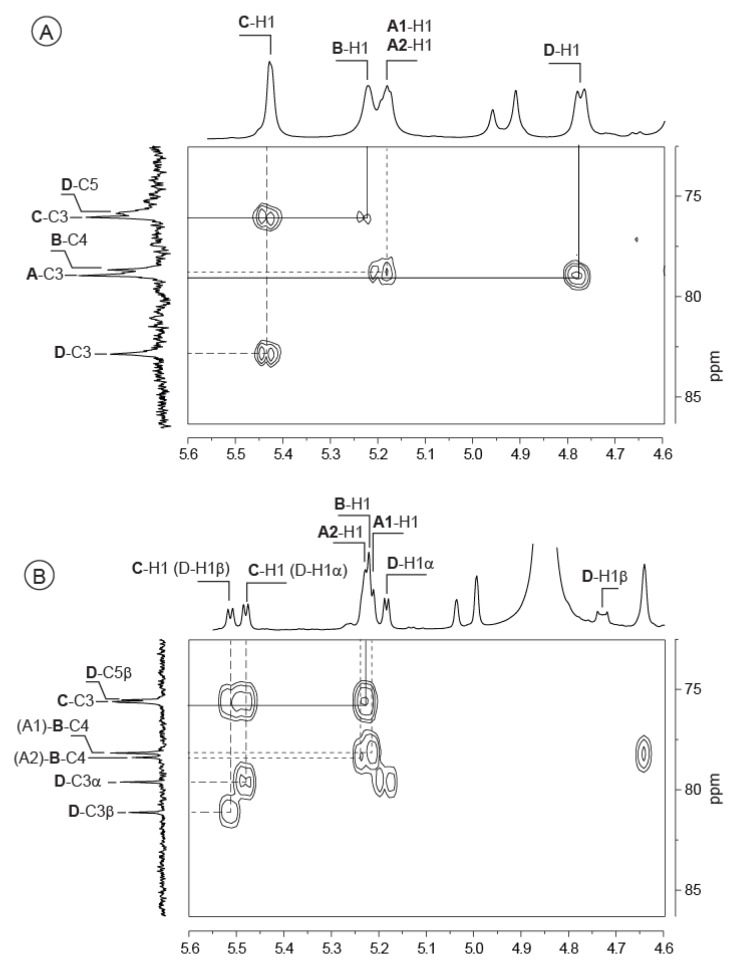

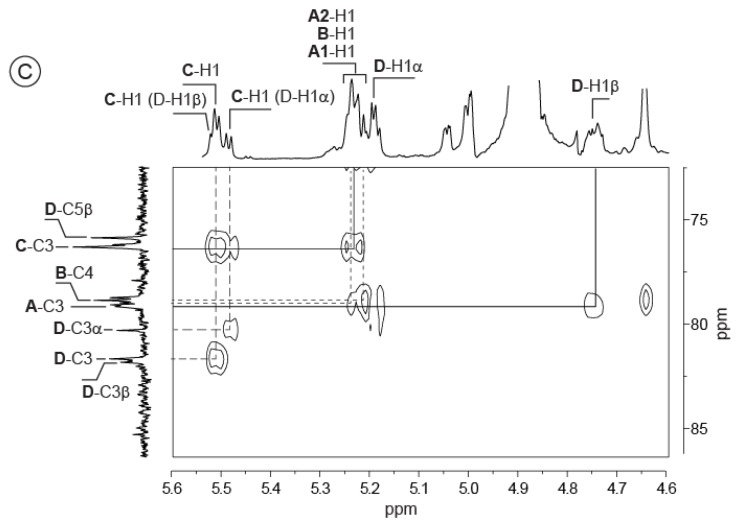
^1^H-^13^C Heteronuclear chemical shift correlated spectrum (HMBC) of the *Vibrio alginolyticus* exopolysaccharide (**A**) and its oligosaccharides: DP4 (**B**) and DP8 (**C**). Correlations demonstrating the linkages between residues in the repetition moieties are indicated.

HMBC experiments to analyze the ^1^H/^13^C heterenuclear coupling relationships highlighted linkage between most of the residues (Figure 3A). The A-C3 carbon (78.97 ppm) was coupled to the D-H1 proton (4.77 ppm), indicating that the β-d-GlcNAc (D) residue is linked to the α-d-GalA (A) residue via a β(1,3) linkage. Similarly, the correlation between B-C4 (78.68/78.85 ppm) and A-H1 (5.18/5.19 ppm) suggested that the linkage between the two α-d-GalA residues A and B occurs through an α(1,4) linkage. The linkage between the two α-d-GalA residues B and C was also demonstrated (correlations C-C3: 76.06/B-H1: 5.22 ppm and B-C1: 97.20/C-H3: 4.03 ppm) and B is linked to C via an α(1,3) linkage. The linkage between the α-d-GalA residue C with the β-GlcNAc (D) could not be determined here.

### 2.3. Preparation and Analysis of Standard Oligosaccharides

**Figure 4:**
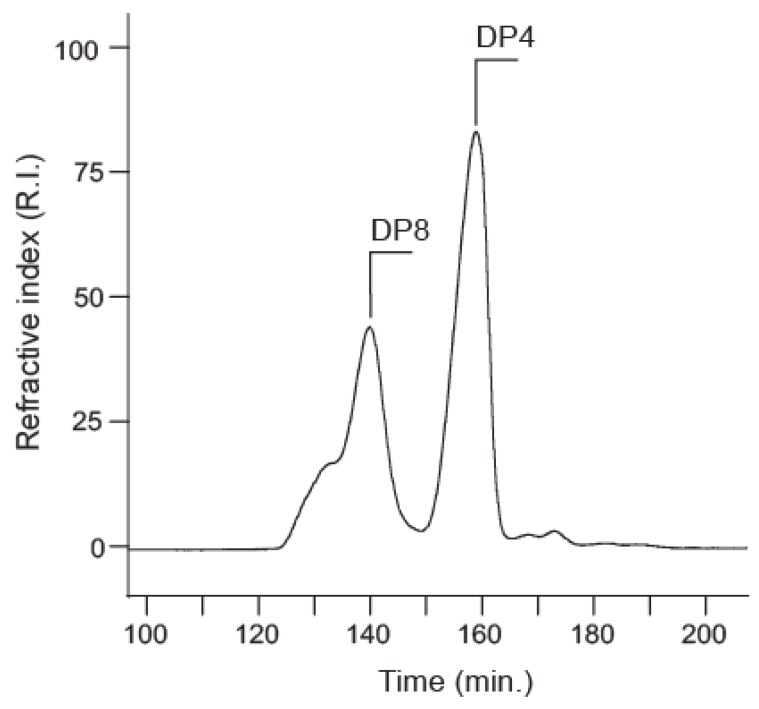
Size-exclusion chromatogram of the acid-hydrolyzed *Vibrio alginolyticus* exopolysaccharide. The two main products, DP4 and DP8, are indicated.

After mild acid hydrolysis, the degradation of the EPS was monitored by SEC (Figure 4). After 20 min in 1 M TFA at 100 °C, degradation led to three main peaks centered at 130 min (shoulder), 140 min and 160 min elution times. ^1^H NMR of the fractions collected at elution times of 150–165 min is given in Figure 2. The spectra showed all the characteristic signals of the polysaccharide, along with additional signals attributed to the reducing and non-reducing ends of the oligosaccharide. The signal of D-H1 (polymer) at 4.77 ppm shifted to 4.73 ppm for D-H1β (DP4). A new D-H1α signal appeared at 5.18 ppm indicating that cleavage occurred at the reducing end of the D residues. The C-H1 (polymer) at 5.43 ppm was split into two doublet signals at 5.48 ppm and 5.51 ppm (DP4). This observation suggested the α-galacturonic residue C was linked to the β-GlcNAc (D) and that the splitting distinguished C(α) from C(β) residues linked to the β- or α-GlcNAc residues, respectively. ^1^H/^13^C HMBC showed the correlation between D-C3 (α/β) (80.37 ppm/81.89 ppm) and C-H1 (Dα/β) (5.48 ppm/5.51 ppm), confirming that residue C is linked to the reducing end of β-GlcNAc (D) via an α(1,3) linkage.

The fractions collected at elution times of 135–145 min contained a single oligosaccharide whose spectra showed similar characteristics to the polymer and the DP4. The α- and β-anomeric protons of the d-GlcNAc were present, as observed for the DP4, at 5.18 ppm and 4.73 ppm. The anomeric protons of the α-d-GalA (C) residues linked to the d-GlcNAc were also present, but signal intensity was different. Integrations of C-H1 (Dα) and C-H1 (Dβ) revealed a 1:2.3 ratio (Figure 2B) instead of a 1:0.6 ratio (Figure 2C) observed for the DP4, suggesting that one C residue was linked to d-GlcNAc (α- and β-anomer) located at the reducing end and one C residue was linked to d-GlcNAc (β-anomer) located within the oligosaccharide. The COSY spectra distinguished α-GalA (A) residues located at the non-reducing end from those linked to d-GlcNAc (the corresponding signals are indicated in Figure 2C). Altogether, integration of the anomeric protons, as well as complete assignment of the proton and carbon chemical shifts (Table 2 and Table 3) confirmed that this oligosaccharide was an octasaccharide made of two DP4 repetition moieties.

### 2.4. Position and Distribution of d-Alanine and d-Serine

The two amino acids, d-alanine and d-serine, detected in the chromatography analyses were also observed by NMR. The proton and carbon chemical shifts were attributed unambiguously using COSY, HMBC and HSQC experiments. The chemical shifts reported in Table 4 are in agreement with known d-alanine and d-serine chemical shifts. The acid treatment and purification steps required to prepare the oligosaccharides DP4 and DP8 did not remove the amino acid, which remained bound to the oligosaccharides; their chemical shifts are reported in Table 4.

**Table 4:** Proton and carbon chemical shifts of the alanine and serine amino acids linked to the carbohydrate backbone. Ala-A1: alanine linked to residue A1; Ala-C: alanine linked to residue C; Ser-A2: serine linked to residue A2.

	VA-EPS: [ABCD]n	DP4: A^nr^BC^r'^D^r^	DP8: A^nr^BCDABC^r′^D^r^
Ala-A1			
H2	4.25	4.27	4.26
H3	1.37	1.41	1.41
C1	179.92	180.44	180.46
C2	51.09	51.29	51.31
C3	18.44	18.43	18.40/18.35
Ala-C			
H2	4.27	4.29	4.28
H3	1.38	1.42	1.42
C1	179.38	179.95/179.92	179.96/179.93
C2	51.09	51.29	51.31
C3	18.91	18.71/18.58	18.69
Ser-A2			
H2	4.37	4.40	4.40
H3	3.85	3.91	3.91
C1	176.26	176.66	176.68
C2	57.19	57.35	57.37
C3	62.99	62.96	62.94

**Figure 5:**
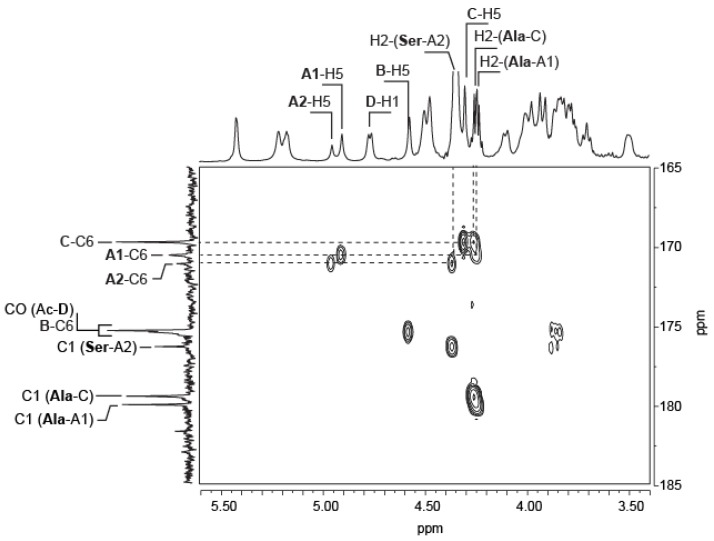
^1^H-^13^C Heteronuclear chemical shift correlated spectrum (HMBC) of the *Vibrio alginolyticus* exopolysaccharide. Correlations between the amino acids (alanine and serine) with the carbohydrate backbone of the polysaccharide are highlighted.

The position of the amino acids on the polysaccharide and on the purified oligosaccharides was deduced from the 2D HMBC spectra. Figure 5 shows a close-up of the HMBC recorded on the polysaccharide highlighting the coupling of the H2 protons of alanine and serine to the C6 carbons involved in the amide linkages (A1-C6, A2-C6 and C-C6). Two different d-alanines were observed, the first was linked to the A1 GalA residue and the second to the C galacturonic acid residue. The d-serine was coupled only to the A2 GalA residue. Integration of the A1-H5 (4.91 ppm) and A2-H5 (4.96 ppm) protons indicated that about 65% of A residues were decorated with d-alanine and other 35% with d-serine.

The position of the amino acids were also confirmed by long-distance ROESY coupling experiments, which showed coupling of the A1-NH proton (7.72 ppm) of the alanine amine to the Ala-A1-H2 and Ala-A1-H3 protons of the d-alanine, and more interestingly to the A1-H5 proton (4.91 ppm). Similar observations on the d-serine amine, with the A2-NH proton (7.83 ppm) coupled to the A2-H5 proton (4.96 ppm), confirmed that the GalA A residue is linked either to d-alanine (A1) or d-serine (A2). A second coupling system for d-alanine was also observed, whereby the C-H5 proton (4.31 ppm) of the GalA residue was coupled to the amine proton of alanine (7.80 ppm), in agreement with an amide linkage of d-alanine on this residue.

## 3. Experimental Section

### 3.1. Production, Isolation and Purification of VA-EPS Exopolysaccharide

VA-EPS was produced by *Vibrio alginolyticus* (CNCM I-4994) in a 30 L fermenter containing marine broth medium (30 g/L sea salts, 1 g/L yeast extracts, 4 g/L peptone) supplemented with glucose (30 g/L) at 25 °C. The culture medium was inoculated at 10% (v/v) with a bacterial suspension in the exponential growth phase. The pH was adjusted and maintained at 7.2 by automatic addition of 1 M NaOH. The medium was oxygenated at 15 L/min with an agitation rate of 350 rpm. After 72 h of fermentation, bacterial cells were removed from the culture medium by centrifugation (16,000× *g*, 30 min). The supernatant, containing the excreted VA-EPS, was then purified by filtration through a cellulose membrane (0.7 μm) and then by ultrafiltration (100 kDa). The sample was freeze-dried and stored at room temperature away from light and moisture.

### 3.2. Monosaccharide Analysis

The molar ratio of monosaccharides was determined according to [9] modified by [10]. 400 μg of EPS were hydrolyzed with 500 μL of 3 M MeOH/HCl at 110 °C for 4 h. After neutralization with silver carbonate a re-*N*-acetylation step was made with 50 μL Ac_2_O overnight at room temperature. After centrifugation, and evaporation of the supernatant, the methyl glycosides were converted to their corresponding trimethylsilyl derivatives by adding 80 μL pyridine and 80 μL of BSTFA:TMCS (99:1; Supelco, Bellefonte, PA, USA). Separation and quantification of the per-*O*-trimethylsilyl methyl glycosides were performed by gas-liquid chromatography (GLC) using an Agilent system equipped with a HP-5ms capillary column (Agilent 0.25 mm × 30 m). The trimethylsilyl derivatives were analyzed using the following temperature program: 120 °C for 1 min, 120 °C → 180 °C at 3 °C/min, 180 °C → 200 °C at 3°C/min, 200 °C for 5 min.

### 3.3. Methylation Analysis

Glycosyl-linkage positions were determined as described in [11]. 20 mg of the native EPS in 10 mL H_2_O were carboxyl-reduced by treatment with 650 mg of *N*-cyclohexyl 1-*N*’[β (*N*-methyl-morpholino)-ethyl] carbodiimide P-toluene sulfonate and with 3 mg of NaBD_4_ for 4 h at room temperature [12]. After dialysis against distilled water and lyophilization, the carboxyl reduced EPS was dissolved in 500 μL DMSO. Hydroxyl groups were then methylated using 500 μL of 2.5 N butyl lithium in hexanes and 500 μL of methyl iodide [13]. Methylation reaction was stopped with 1 mL H_2_O and the methylated compounds were extracted with CH_2_Cl_2_. The methylated products were then hydrolyzed in 500 μL of 2 M TFA for 2 h at 120 °C. After evaporation of TFA the corresponding monomers were reduced with 20 mg of NaBD_4_ in a NH_4_OH solution for 30 min at 80 °C, and finally acetylated with 200 μL of 1-methyl imidazole and 2 mL of pyridine for 10 min at room temperature. GLC-mass spectrometry (MS) was performed on an Agilent instrument fitted with a HP-5ms capillary column (Agilent, Santa Clara, CA, USA, 0.25 mm × 30 m). The temperature program was 90 °C for 1 min, 90 °C → 300 °C at 5 °C/min, 300 °C for 1 min. Ionization was carried out in electron impact mode (EI, 70 eV).

### 3.4. Amino Acid Composition

VA-EPS (1% w/v) in water was hydrolyzed by addition of an equal volume of 11 N HCl for 5 h at 105 °C. After a cooling step, neutralization was carried out with 10 N NaOH, then with 1 N NaOH, using pH-indicator paper to reach pH 6–7. The final volume was adjusted to 25 mL with ultrapure water and filtered on a 0.2 μm sterilizing grade membrane.

The neutralized samples and an amino acid hydrolysate standard (Waters, Milford, MA, USA) were derivatized by adding 6-aminoquinolyl-*N*-hydroxysuccinimidyl carbamate using AccQ•Tag™ Ultra Derivatization kit (Waters, Milford, MA, USA). The derivatized samples (10 μL) were injected on a AccQ Tag Ultra C18 column (1.7 μm 2.1 × 100 mm, Waters, Milford, MA, USA) mounted on a Waters Acquity H class ultra-high-performance liquid chromatography apparatus equipped with UV detector working at 260 nm.

A gradient elution was conducted for 10 min at 55 °C starting with 100% eluent A (acetonitrile/formic acid) to 100% of eluent B (acetonitrile). Signals were compared to derivatized amino acid standards. According to experimental conditions, serine and alanine eluted at 2.3 min and 4.4 min, respectively.

### 3.5. Sulfate Content

Elemental analysis was performed by the CNRS Institute of Analytical Sciences (Lyon, France). Potential sulfate content (sodium salt) was evaluated.

### 3.6. Molecular Weight Determination

The molecular weight of VA-EPS was determined by high-performance size-exclusion chromatography (HPSEC) using an eighteen-angle light scattering detector, coupled with refractive index and specific refractive index increment dn/dc (DAWN™ HELEOS, Wyatt, Santa Barbara, CA USA). Elution was performed on Shodex OHpak SB-805 HQ and OHpak SB-806 HQ placed in series (Phenomenex, fractionation range <2.10^7^ g/mol) with 0.1 M NaNO_3_ as the eluent. To calculate the molecular mass, the dn/dc value used was 0.145 mL/g. The polydispersity index was calculated from the Mw/Mn ratio.

### 3.7. Acid Hydrolysis and Oligosaccharides Purification

VA-EPS underwent mild acid hydrolysis, 20 min in 1 M TFA at 100 °C. The resulting oligosaccharides were fractionated by SEC on a Toyopearl HW-40 (Tosoh, fractionation range <10^4^ Da) with 0.1 M NaCO_3_ as the eluent.

### 3.8. NMR

Carbon-13 and proton NMR spectra were recorded with a Bruker Avance 400 spectrometer operating at a frequency of 100.618 MHz for ^13^C and 400.13 MHz for ^1^H. Samples were solubilized in D_2_O at a temperature of 293 K for the oligosaccharides and 353 K for the polysaccharide. Residual signal of the solvent was used as internal standard: HOD at 4.85 ppm at 293 K and 4.25 at 353 K. ^13^C spectra were recorded using 90° pulses, 20,000 Hz spectral width, 65,536 data points, 1.638 s acquisition time, 1 s relaxation delay and between 8192 and 16,834 scans. Proton spectra were recorded with a 4006 Hz spectral width, 32,768 data points, 4.089 s acquisition times, 0.1 s relaxation delays and 16 scans. The ^1^H and ^13^C NMR assignments were based on ^1^H-^1^H homonuclear and ^1^H-^13^C heteronuclear correlation experiments (correlation spectroscopy, COSY; heteronuclear multiple-bond correlation, HMBC; heteronuclear multiple quantum correlation, HMQC). They were performed with a 4006 Hz spectral width, 2048 data points, 0.255 s acquisition time, 1 s relaxation delay; from 32 up to 512 scans were accumulated.

## 4. Conclusions

The structure of the exopolysaccharide secreted by *Vibrio alginolyticus* (CNCM I-4994) was completely resolved. It is a linear polysaccharide made up of two residues: *N*-acetyl-glucosamine and galacturonic acid, whose repetition unit—according to linkage and decoration—is a tetrasaccharide. During structural analyses of the polysaccharide, we observed that acid hydrolysis produced an oligosaccharide series having a size equal to (DP4) or a simple multiple (DP8) of the repetition unit, suggesting strong reactivity differences between the glycosidic linkages of the polysaccharide. A second, very interesting feature was the discovery of amino acids naturally bound to uronic residues via amide linkages. Several organic derivatives (pyruvate, succinic acid, acetate) or inorganic acid (sulfate, phosphate) have already been found in many EPSs. However, decoration of the carbohydrate backbone by one amino acid bond *via* an amide linkage appeared until now, limited to *O*-antigen lipopolysaccharides [14,15,16] or capsular polysaccharides [17,18]. Therefore, to our knowledge, this is the first report of a polysaccharide secreted by a Gram-negative bacteria carrying two different amino acids (alanine and serine).
